# 

**Published:** 1895-12

**Authors:** 


### INDEX TO VOLUME XVI.

A.

Academy of Stomatology, 39, 165, 240, 306, 372, 770.
A Cautionary Signal, 522.
A Compend of Dental Prosthesis and Metallurgy, 189.
A Compend of Materia Medica, Therapeutics, and Prescription Writing, with Especial Reference to the Physiological Action of Drugs, 192.
A Consideration of Some Problems in a Dentist's Life, 486.
A Contribution to the Study of the Structure of the Dentine; the so-called "Sheaths of Neumann," 655.
Additions to Dr. Potter's Paper, 131.
After-Pains from Extraction of Exostosed Teeth, 651.
A Greater Need for Professional Dignity, 379.
Allan, George S., Notes on Hydrogen Dioxide ($H_2O_2$), 204.
Allen, Harrison, Diseases of the Maxillary Sinus, 261.
Alumni Association, University of Michigan, 526.
Alveolaris, Pyorrhœa, 591.
Amalgam, Glass Fillings, Jacket Crowns, Gold Caps, Bridges, etc.—A Scrap of their History, 353.
Amalgams, Some Principles relating to, 214.
American Academy of Dental Science, 33, 292, 358, 690, 763.
American Dental Association, 386, 460, 523, 611.
American Dental Society of Europe, 462, 654.
A Method of filling Root-Canals, 31.
A Method of inserting Gold Fillings with the Use of Hand-Burnishers, as practised exclusively for Seventeen Years, 325.
An Address upon the Fiftieth Memorial Celebration of the Discovery of Anæsthesia, 20.
Anæsthesia, An Address upon the Fiftieth Memorial Celebration of the Discovery of, 20.
  A New Apparatus for continuing, 731.
  History of the Discovery of Modern, 1.
A National Dental Museum, 555.
Andrews, R. R. A Contribution to the Study of the Structure of the Dentine,—the so-called "Sheaths of Neumann," 655.
An Electrical Disinfectant, 65.
A New Apparatus for continuing Anæsthesia while operating in the Mouth, 731.
A New Method of reducing Dislocation of the Jaw, 257.
Annual Meeting of the Harvard Odontological Society, 320.
Annual Meeting of the International Dental Publication Company, 192.
Answers, Questions and, 453.
Antisepsis in Dentistry, 62.
A Plea for Enthusiasm, 62.
A Plea for Moderation, 547.
Asbury Park and the Conventions, 381.
Assistance and Assistants, 736.

B.

Bacteria, Plastics as a Bar to, 349.
Bad Luck, 651.
Balsamo del Deserto, Further Experience with, 601.
Bar to Bacteria, Plastics as a, 349.
Baxter, Edwin C., 648.
Bibliography: A Compend of Dental Prosthesis and Metallurgy, 189.
  A Compend of Materia Medica, Therapeutics, and Prescription-Writing, with Especial Reference to the Physiological Action of Drugs, 192.
  Brook Farm. Historic and Personal Memoirs, 190.
  Catching's Compendium of Practical Dentistry for 1894, 317.
  Dental Medicine: A Manual of Dental Materia Medica and Therapeutics, 316.
  Descriptive Anatomy of the Human Teeth, 318.
  General Surgery and Pathology for Dentists, 647.
  Manual of Operative Technics, 186.
  Methoden und Neuerungen auf dem Gebiete der Zahnheilkunde. Von Wilh. Herbst, Zahnarzt in Bremen. Verlag Odontologische Verlagsanstalt. Berlin, 782.
  Sammlung von Mikrophotographien zur Veranschaulichung der Mikroscopischen Struktur der Zähne des Menschen. Herausgegeben von Dr. Dent. Surg. A. Gysi und Dr. Med. C. Röse. Zürich, Schweiz. (Micro-

BIBLIOGRAPHY:
photographs of Dental Histology.) 187.
Transactions of the American Dental Association at the Thirty-third and Thirty-fourth Annual Sessions, 521.
Transactions of the Midwinter Fair Dental Congress, 521.
Transactions of the Twenty-fifth Annual Session of the Southern Dental Association, 521.
Transactions of the World's Columbian Dental Congress, 315.
Useful Hints for the Busy Dentist, 520.
World's History and Review of Dentistry, 519.
Bites, Mosquito, 651.
Black, G. V. Descriptive Anatomy of the Human Teeth, 318.
Bleaching Teeth by Electricity, 213.
Block-Teeth, Carving of, 284.
Bödecker's Book, Reply to Review of, 319.
Brackett, C. A., Assistance and Assistants, 736.
Bradley, Frederick, Judgment an Important Factor in the Preservation of Teeth, 664.
Bridges, Glass Fillings, Jacket Crowns, Amalgam, Gold Caps, etc.—A Scrap of their History, 353.
Broken Plaster Casts, To mend, 651.
Brook Farm. Historic and Personal Memoirs, 190.
Broomell, I. Norman. Reply to Dr. W. H. Trueman, 543.
Brown, Augustus Woodruff, 588.
Burchard, Henry H. Cause and Being of Teeth, 670.
The Rational Therapeutics of Pyorrhœa Alveolaris, 477.

C.

Cancer of the Tongue, 146.
Carving of Block-Teeth, 284.
Case, Calvin S. Dental and Facial Orthopædia, 463.
Catching, B. H. Catching's Compendinm of Practical Dentistry for 1894, 317.
Catching's Compendium of Practical Dentistry for 1894, 317.
Cause and Being of Teeth, 670.
Central Dental Association of Northern New Jersey, 117.
Chandler, Thomas H., 718.
Chicago Dental Society, 384.
Chloride of Ethyl, The Application of, 196.
Cleaning Impression Trays, Simple Method of, 523.
Coagulants, The Relative Penetrating Power of, 11.
Codman, John Thomas. Brook Farm. Historic and Personal Memoirs, 190.
Coney, F. A. Carving of Block-Teeth, 284.
Connecticut Valley Dental Society, 63.
Contour, On, 288.
Conventions, Asbury Park and the, 381.
Conventions, The Annual, 455.
Conventions, The Recent, 641.
Cravens, J. E. Pyorrhœa Alveolaris, 591.
Culture, True, 196.
CURRENT NEWS: Alumni Association, University of Michigan, 526.
American Dental Association, 386, 460, 523, 611, 679.
American Dental Society of Europe, 462, 654.
Annual Meeting of the Harvard Odontological Society, 320.
Annual Meeting of the International Dental Publication Company, 192.
Chicago Dental Society, 384.
Connecticut Valley Dental Society, 63.
Dental Association of New South Wales, 653.
Dental Society of the State of New York, 322, 526.
First District Dental Society of New York, 195.
Harvard Dental Alumni Association, 524.
Illinois State Dental Society, 322, 525.
Massachusetts Dental Society, 386, 786.
Missouri State Dental Association, 384.
National Association of Dental Examiners, 322, 385, 638.
National Association of Dental Faculties, 462, 709.
New Jersey State Dental Society, 385.
Pennsylvania State Dental Examining Board, 385.
Pennsylvania State Dental Society, 461.
Report of Treasurer of World's Columbian Dental Congress, 783.
Southern Dental Association, 654.
Souvenir Volume of the Wells Celebration, 131.
The Horace Wells Permanent Memorial, under the Auspices of the American Dental Association, 194.
To arrange for Interstate Meeting, Missouri, 462.
Tri-State Meeting, 258.
Union Dental Meeting, 653.
Union Meeting, 259.
Woman's Dental Association, 63, 387, 525.
Curtis, G. Lenox. Theory and Results, 419.
Cusps of the Human Molar Teeth, The History of the, 389.

D.

Daly, James H. A Method of Filling Root-Canals, 31.
The Thorough Removal of Deposits in the Successful Treatment of Pyorrhœa, 605.
Darby, Edwin T. Gouty Pericementitis, 197.

Davenport, S. E. The Correction of Injury resulting from Extraction, 81.
Deformities of the Hard Palate in Degenerates, 719.
Degrees, The Faculties and Honorary, 646.
Denial, Self-, 651.
Dental and Facial Orthopædia, 463.
Dental Association of New South Wales, 653.
Dental Associations, Politics in, 124.
Dental Colleges, Entrance Examinations to, 184.
Dental Digest, 127, 256.
Dental Engine, Method of mounting Disks and Points for, 652.
Dental Faculties, The National Association of, 713.
Dental Furnaces, 279.
Dental Medicine: A Manual of Dental Materia Medica and Therapeutics, 316.
Dental Practice, The Scientific Spirit and the Ethics of, 343.
Dental Prosthesis and Metallurgy, A Compend of, 189.
Dental Services, System of charging for, 589.
Dental Society of the State of New York, 322, 526.
Dental Training, 586.
Dentistry and Corsets, 62.
Dentistry in the Public Schools, 482.
Dentistry, Specialism in, 253.
Dentists, General Surgery and Pathology for, 647.
Dentist's Life, A Consideration of Some Problems in a, 486.
Dentists, The Census of 1890 and the, 551.
Descriptive Anatomy of the Human Teeth, 318.
Devitalization of Highly Inflamed Pulp, 651.
Digest, The Dental, 127, 256.
Dignity, Professional, A Greater Need for 379.
Diiodoform, 387.
Discovery of Anæsthesia, An Address upon the Fiftieth Memorial Celebration of the, 20.
Diseases of the Maxillary Sinus, 261.
Diseases of the Teeth, Some Disturbances caused by, 266.
Disinfectant, An Electrical, 65.
Dislocation of the Jaw, A New Method of Reducing, 132, 257.
Domestic Correspondence: Additions to Dr. Potter's Paper, 131.
A New Method of Reducing Dislocation of the Jaw, 257.
Reply to Dr. Max Greenbaum, 128.
Reply to Review of Bödecker's Book, 319.
Dr. Max Greenbaum, A Reply to, 128.
Dr. Potter's Paper, Additions to, 131.
Dr. W. H. Trueman, Reply to, 543.

E.

Editorial: A Greater Need for Professional Dignity, 379.
Asbury Park and the Conventions, 381.
Dental Training, 586.
Entrance Examinations to Dental Colleges, 184.
Erratum, 647.
Explanation, 256.
Golden Anniversary Celebration, 782.
Pennsylvania State Dental Society, 456.
Politics in Dental Associations, 124.
Proof-Reader gone Wrong, 127.
Questions and Answers, 453.
Specialism in Dentistry, 253.
Spurious History, 777.
The Annual Conventions, 455.
The Close of Volume XVI., 780.
The Dental Digest, 127, 256.
The Faculties and Honorary Degrees, 646.
The Fiftieth Memorial Celebration, 58.
The Loyalty of Society Members, 182.
The National Association of Dental Faculties, 713.
The Recent Conventions, 641.
The Souvenir Volume of the Wells Celebration, 127.
The Wrong Use of Words, 313.
Will Dentistry be absorbed by Medicine? 517.
Electricity, Bleaching Teeth by, 213.
Entrance Examinations to Dental Colleges, 184.
Errata, 61.
Erratum, 647.
Essig, C. J., The Scientific Spirit and the Ethics of Dental Practice, 343.
Examinations, Entrance, to Dental Colleges, 184.
Exostosed Teeth, After-Pains from Extraction of, 651.
Extraction, The Correction of Injury resulting from, 81.

F.

Facial Orthopædia, 463.
Fillebrown, Thomas. History of the Discovery of Modern Anæsthesia, 1.
A New Apparatus for Continuing Anæsthesia while operating in the Mouth, 731.
Filling, Root-Canal, 652.
First District Dental Society of New York, 195.
Foreman, J. W. Impacted Third Molors, 475.
Furnaces, Dental, 279.
Further Experience with Balsamo del Deserto, 601.

G.

Garretson, James E. An Address upon the Fiftieth Memorial Celebration of the Discovery of Anæsthesia, 20.

General Surgery and Pathology for Dentists, 647.
Glass Fillings, Jacket Crowns, Amalgam, Gold Caps, Bridges, etc.—A Scarp of their History, 353.
Gold Fillings, a Method of inserting, with the Use of Hand-Burnishers, as practised exclusively for Seventeen Years, 325.
Golden Anniversary Celebration, 782, 786.
Gone Wrong, Proof-Reader, 127.
Gorgas, Ferdinand J. S. Dental Medicine: a Manual of Dental Materia Medica and Therapeutics, 316.
Gouty Pericementitis, 197.
Greenleaf, Robert W. The Relation of Modern Therapeutics to the Practice of Dentistry, 152.

H.

Hard Palate, Deformities of, in Degenerates, 719.
Hardened Rubber, Restoration of, 652.
Harlan, A. W. Transactions of the World's Columbian Dental Congress, 315.
Harvard Dental Alumni Association, 524.
Harvard Odontological Society, 104.
Hawes, Arnold C., 457.
Head, Joseph. Plastics as a Bar to Bacteria, 349.
Historic and Personal Memoirs. Brook Farm, 190.
History of the Discovery of Modern Anæsthesia, 1.
Holmes, E. Proctor. Dentistry in the Public Schools, 482.
Honorary Degrees, The Faculties and, 646.
Human Teeth, Descriptive Anatomy of the, 318.
Huntington, T. G. On Contour, 288.
Hydrogen Dioxide, Note on, 78, 204.
Hypnotism, The Physiology of, 27.

I.

Illinois State Dental Society, 322, 525.
Impacted Third Molars, 475.
Impression Trays, Simple Method of Cleaning, 523.
Inflamed Pulp, Devitalization of, 651.
Injury resulting from Extraction, The Correction of, 81.

J.

Jacket Crowns, Glass Fillings, Amalgam, Gold Caps, Bridges, etc.—A Scrap of their History, 353.
Judgment an Important Factor in the Preservation of Teeth, 664.

K.

Kirk, Edward C. Some Principles relating to Amalgams, 214.
Kratzer, C. V. The Heroic in Dental Operations, as contrasted with Conservatism, 744.

L.

Leffmann, Henry. Note on Hydrogen Dioxide as furnished to Practising Dentists, 78.
Lennmalin, Herman. World's History and Review of Dentistry, 519.
Libby, Henry F. A Method of inserting Gold Fillings with the Use of Hand-Burnishers, as practised exclusively for Seventeen Years, 325.
Luck, Bad, 651.

M.

Manual of Operative Technics, 186.
Massachusetts Dental Society, 386, 786.
Maxillary Sinus, Diseases of, 261.
Meeting, Tri-State, 258.
Meeting, Union, 259.
Memorial Celebration of the Fiftieth Anniversary of the Discovery of Anæsthesia, 50.
Metallurgy and Dental Prosthesis, A Compend of, 189.
Method of mounting Disks and Points for Dental Engine, 652.
Missouri State Dental Association, 384, 608.
Modern Anæsthesia, History of the Discovery of, 1.
Moderation, A Plea for, 547.
Moderation in Practice and in Statement, 133.
Modern Therapeutics, The Relation of, to the Practice of Dentistry, 152.
Molars, Impacted Third, 475.
Molar Teeth, The History of the Cusps of the Human, 389.
Mosquito Bites, 651.
Museum, A National Dental, 555.
Musgrove, Thomas H., 458.

N.

National Association of Dental Examiners, 322, 385, 638.
National Association of Dental Faculties, 462, 709.
New Jersey State Dental Society, 385.
New York Institute of Stomatology, 440.
New York Odontological Society, 84, 160, 223, 429, 491, 556, 752.
Noble, H. B. A National Dental Museum, 555.
Note on Hydrogen Dioxide as furnished to Practising Dentists, 78.
NOTES AND COMMENTS: A Cautionary Signal, 522.
- After-Pains from Extraction of Exostosed Teeth, 651.
- Antisepsis in Dentistry, 62.
- A Plea for Enthusiasm, 62.
- Bad Luck, 651.
- Dentistry and Corsets, 62.
- Devitalization of Highly Inflamed Pulp, 651.
- Errata, 61.

Notes and Comments:
Method of Mounting Disks and Points for Dental Engine, 652.
Mosquito Bites, 651.
Restoration of Hardened Rubber, 652.
Root-Canal Filling, 652.
Rubber Dam for Pulp-Capping, 652.
Self-Denial, 651.
Simple Method of Cleaning Impression Trays, 523.
System of Charging for Dental Services, 589.
Teacher and Taught, 590.
The Applieation of Chloride of Ethyl, 196.
The Dentist's Duty to himself and his Clients, 590.
The Necessity of Training, 61.
To Mend Broken Plaster Casts, 651.
True Culture, 196.
Notes on Hydrogen Dioxide ($H_2O_2$), 204.

O.

Obituary: Baxter, Edwin C., 648.
Brown, Augustus Woodruff, 588.
Chandler, Thomas H., 718.
Hawes, Arnold C., 457.
Musgrove, Thomas H., 458.
Patrick, John J. R., 382.
Resolutions on the Death of, 522.
Tomes, Sir John, 649.
Tucker, Elisha G., 459.
Weil, Ludwig Adolf, 383.
Odontological Society of Pennsylvania, 100, 302, 369, 514, 577, 709.
On Contour, 288.
Opening Root-Canals, Sulphuric Acid for, 259.
Operative Technics, Manual of, 186.
Original Communications: A Consideration of Some Problems in a Dentist's Life, 486.
A Contribution to the Study of the Structure of the Dentine; the So-called "Sheaths of Neumann," 655.
A Method of filling Root-Canals, 31.
A Method of inserting Gold Fillings with the Use of Hand-Burnishers, as practised exclusively for Seventeen Years, 325.
An Address upon the Fiftieth Memorial Celebration of the Discovery of Anæsthesia, 20.
A National Dental Museum, 555.
An Electrical Disinfectant, 65.
A Plea for Moderation, 547.
Bleaching Teeth by Electricity, 213.
Cancer of the Tongue, 146.
Carving of Block-Teeth, 284.
Cause and Being of Teeth, 670.
Dental and Facial Orthopædia, 463.
Dental Furnaces, 279.
Dentistry in the Public Schools, 482.

Original Communications:
Diseases of the Maxillary Sinus, 261.
Further Experience with Balsamo del Deserto, 601.
Glass Fillings, Jacket Crowns, Amalgam, Gold Caps, Bridges, etc.—A Scrap of their History, 353.
Gouty Pericimentitis, 197.
History of the Discovery of Modern Anæsthesia, 1.
Impacted Third Molars, 475.
Judgment an Important Factor in the Preservation of Teeth, 664.
Moderation in Practice and in Statement, 133.
Note on Hydrogen Dioxide as furnished to Practising Dentists, 78.
Notes on Hydrogen Dioxide ($H_2O_2$), 204.
On Contour, 288.
Plastics as a Bar to Bacteria, 349.
Pyorrhœa Alveolaris, 591.
Reply to Dr. W. H. Trueman, 543.
Salol, 472.
Some Disturbances caused by Diseases of the Teeth, 266.
Some Principles relating to Amalgams, 214.
Specialism, 270.
Sulphuric Acid and Peroxide of Sodium in the Treatment of Pulpless Teeth, 74.
Syphilis, with Special Reference to the Relations of the Dental Profession to the Disease, 536.
The Application of Some Principles of Law to the Practice of Dentistry, 403.
The Census of 1890 and the Dentists, 551.
The Correction of Injury resulting from Extraction, 81.
The History of the Cusps of the Human Molar Teeth, 389.
Theory and Results, 419.
The Physiology of Hypnotism, 27.
The Rational Therapeutics of Pyorrhœa Alveolaris, 477.
The Relation of Modern Therapeutics to the Practice of Dentistry, 152.
The Relative Penetrating Power of Coagulants, 11.
The Scientific Spirit and the Ethics of Dental Practice, 343.
The Thorough Removal of Deposits in the Successful Treatment of Pyorrhœa, 605.
What has been done by the Profession in France in Pyorrhœa Alveolaris, 660.
Which Method of Root-Canal Filling will completely obliterate Space, 527.
Orthopædia, Dental and Facial, 463.
Osborn, Henry Fairfield. The History of the Cusps of the Human Molar Teeth 389.

P.

Patrick, John J. R., 382.
Resolutions on the Death of, 522.
Peirce, C. N. What has been done by the Profession in France on Pyorrhœa Alveolaris, 660.
Pennsylvania State Dental Examining Board, 385.
Pennsylvania State Dental Society, 456, 461.
Pericementitis, Gouty, 197.
Peroxide of Hydrogen Preparations, Value of, 323.
Perry, Safford G. Moderation in Practice and in Statement, 133.
Which Method of Root-Canal Filling will completely obliterate Space, 527.
Peterson, Frederick, Deformities of the Hard Palate in Degenerates, 719.
Plastics as a Bar to Bacteria, 349.
Politics in Dental Associations, 124.
Porter, Alfred H. The Physiology of Hypnotism, 27.
Potter, Samuel O. A Compend of Materia Medica, Therapeutics, and Prescription Writing, with Especial Reference to the Physiological Action of Drugs, 192.
Practice, Moderation in, 133.
Practice of Dentistry, The Application of Some Principles of Law to, 403.
Practice of Dentistry. The Relation of Modern Therapeutics to, 152.
Preservation of Teeth, Judgment an Important Factor in, 664.
Professional Dignity, A Greater Need for, 379.
Profession in France on Pyorrhœa Alveolaris, What has been done by the, 660.
Proof-Reader gone Wrong, 127.
Public Schools, Dentistry in the, 482.
Pulp-Capping, Rubber Dam for, 652.
Pulsford, Henry A. Syphilis, with Special Reference to the Relations of the Dental Profession to the Disease, 536.
Purrington, W. A. The Application of Some Principles of Law to the Practice of Dentistry, 403.
Pyorrhœa Alveolaris, 591.
The Rational Therapeutics of, 477.
What has been done by the Profession in France on, 660.
Pyorrhœa, The Thorough Removal of Deposits in the Successful Treatment of, 605.

Q.

Questions and Answers, 453.

R.

Recent Patents, 64.
Reducing Dislocation of the Jaw, A New Method of, 132.
Reply to Dr. Max Greenbaum, 128.
Reply to Dr. W. H. Trueman, 543.
Reply to Review of Bödecker's Book, 319.
Report of Treasurer of World's Columbian Dental Congress, 783.
Resolutions on the Death of J. J. R. Patrick, 522.
Restoration of Hardened Rubber, 652.
Results, Theory and, 419.
Review of Dentistry, World's History and, 519.
Root-Canals, A Method of Filling, 31.
Filling, 652.
Filling of, 527.
Sulphuric Acid for opening, 259.
Roughton, Edmund W. General Surgery and Pathology for Dentists, 647.
Rubber Dam for Pulp-Capping, 652.

S.

Salol, 472.
Sanger, R. M. Salol, 472.
Schools, Dentistry in the Public, 482.
SELECTIONS: A New Method of reducing Dislocation of the Jaw, 132.
Diiodoform, 387.
Sulphuric Acid for opening Root-Canals, 259.
The Value of Peroxide of Hydrogen Preparations, 323.
Self-Denial, 651.
Sharp, W. M. Dental Furnaces, 279.
"Sheaths of Neumann," the so-called: A Contribution to the Study of the Structure of the Dentine, 655.
Simple Method of Cleaning Impression Trays, 523.
Smith, B. Holly. A Plea for Moderation, 547.
SOCIETY MEETINGS: Academy of Stomaatology, 39, 165, 240, 306, 372, 770.
American Academy of Dental Science, 33, 292, 358, 690, 763.
Central Dental Association of Northern New Jersey, 117.
Harvard Odontological Society, 104.
Memorial Celebration of the Fiftieth Anniversary of the Discovery of Anæsthesia, 50.
National Association of Dental Faculties, 709.
New York Institute of Stomatology, 440.
New York Odontological Society, 84, 160, 223, 429, 491, 556, 752.
Odontological Society of Pennsylvania, 100, 302, 369, 514, 577, 709.
Society Members, The Loyalty of, 182.
Some Disturbances caused by Diseases of the Teeth, 266.
Some Principles of Law, The Application of, to the Practice of Dentistry, 403.
Some Principles relating to Amalgams, 214.
Some Problems in a Dentist's Life, A Consideration of, 486.
Southern Dental Association, 654.
Souvenir Volume of the Wells Celebration, 131.
Specialism, 270.
Specialism in Dentistry, 253.

Spurious History, 777.
Statement, Moderation in, 133.
8teele, William H. Useful Hints for the Busy Dentist, 520.
Stevens, Edgar F. Some Disturbances caused by Diseases of the Teeth, 266.
Stevens, E. W. Cancer of the Tongue, 146.
Successful Treatment of Pyorrhœa, The Thorough Removal of Deposits in the, 605.
Sulphuric Acid and Peroxide of Sodium in the Treatment of Pulpless Teeth, 74.
Sulphuric Acid for opening Root-Canals, 259.
Syphilis, with Special Reference to the Relations of the Dental Profession to the Disease, 536.
System of charging for Dental Services, 589.

T.

Taft, Ezra F. A Consideration of Some Problems in a Dentist's Life, 486.
Taught, Teacher and, 590.
Teacher and Taught, 590.
Teeth, Cause and Being, 670.
Teeth, Descriptive Anatomy of the Human, 318.
Teeth, The History of the Cusps of the Human Molar, 389.
The Annual Conventions, 455.
The Application of Chloride of Ethyl, 196.
The Application of Some Principles of Law to the Practice of Dentistry, 403.
The Busy Dentist, Useful Hints for, 520.
The Census of 1890 and the Dentists, 551.
The Close of Volume XVI., 780.
The Correction of In ury resulting om Extraction, 81.
The Dental Digest, 127, 256.
The Dentist's Duties to Himself and his Clients, 589.
The Faculties and Honorary Degrees, 646.
The Fiftieth Memorial Celebration, 58.
The Heroic in Dental Operations, as contrasted with Conservatism, 744.
The History of the Cusps of the Human Molar Teeth, 389.
The Horace Wells Permanent Memorial, under the Auspices of the American Dental Association, 194.
The Loyalty of Society Members, 182.
The National Association of Dental Faculties, 713.
The Necessity of Training, 61.
Theory and Results, 419.
The Physiology of Hypnotism, 27.
The Rational Therapeutics of Pyorrhœa Alveolaris, 477.
The Recent Conventions, 641.
The Relation of Modern Therapeutics to the Practice of Dentistry, 152.
The Relative Penetrating Power of Coagulants, 11.
The Scientific Spirit and the Ethics of Dental Practice, 343.
The Souvenir Volume of the Wells Celebration, 127.
The Thorough Removal of Deposits in the Successful Treatment of Pyorrhœa, 605.
The Value of Peroxide of Hydrogen Preparations, 323.
The Wrong Use of Words, 313.
Third Molars, Impacted, 475.
To arrange for Interstate Meeting, Missouri, 462.
To mend Broken Plaster Casts, 651.
Tomes, Sir John, 649.
Tongue, Cancer of, 146.
Training, Dental, 586.
Transactions of the American Dental Association at the Thirty-third and Thirty-fourth Annual Sessions, 521.
Transactions of the Midwinter Fair Dental Congress, 521.
Transactions of the Twenty-fifth Annual Session of the Southern Dental Association, 521.
Transactions of the World's Columbian Dental Congress, 315.
Treatment of Decay in Approximate Surfaces of Bicuspids and Molars, 747.
Tri-State Meeting, 258.
Treatment of Pulpless Teeth, Sulphuric Acid and Peroxide of Sodium in, 74.
True Culture, 196.
Trueman, Wm. H. Glass Fillings, Jacket Crowns, Amalgam, Gold Caps, Bridges, etc.—A Scrap of their History, 353.
Truman, James. The Relative Penetrating Power of Coagulants, 11.
Tucker, Elisha G., 459.
Twilley, Wm. Shaw. The Census of 1890 and the Dentists, 551.

U.

Union Dental Meeting, 653.
Union Meeting, 259.
University of Michigan Alumni Association, 526.
Useful Hints for the Busy Dentist, 520.
Use of Words, The Wrong, 313.

V.

Van Woert, F. T. Sulphuric Acid and Peroxide of Sodium in the Treatment of Pulpless Teeth, 74.

W.

Warren, Geo. W. A Compend of Dental Prosthesis and Metallurgy, 189.
Weeks, Thomas E. Manual of Operative Technics, 186.
Weil, Ludwig Adolf, 383.
Wells Celebration, Souvenir Volume of the, 131.

Werner, J. G. W. Treatment of Decay in Approximate Surfaces of Bicuspids and Molars, 747.
Westlake, Albert. Bleaching Teeth by Electricity, 213.
What has been done by the Profession in France in Pyorrhœa Alveolaris, 660.
Which Method of Root-Canal Filling will completely obliterate Space, 527.
White, W. H. Further Experience with Balsamo del Deserto, 601.
Will Dentistry be absorbed by Medicine? 517.
Woman's Dental Association, 63, 387, 525.
Wood, Nathan E. Specialism, 270.
Woolf, Albert E. An Electrical Disinfectant, 65.
Words, The Wrong Use of, 313.
World's Columbian Dental Congress, Transactions of, 315.
World's History and Review of Dentistry 519.

SUBSCRIBE FOR THE

### International Dental Journal,

EDITED BY JAMES TRUMAN, D.D.S.

### Published by the International Dental Publication Company.

LOUIS JACK, PRESIDENT, Philadelphia.
BENJAMIN LORD, VICE-PRESIDENT, New York.
C. N. PEIRCE, TREASURER, Philadelphia.
GEO. S. ALLAN, SECRETARY, 51 West Thirty-seventh St., New York.

**Publication Office, 716 Filbert St., Philadelphia, Pa.**

### STOCKHOLDERS.

| | |
|---|---|
| GEORGE EMORY ADAMS | South Orange, N.J. |
| GEO. S. ALLAN | New York, N.Y. |
| R. R. ANDREWS | Cambridge, Mass. |
| H. A. BAKER | Boston, Mass. |
| A. E. BALDWIN | Chicago, Ill. |
| W. C. BARRETT | Buffalo, N.Y. |
| *A. P. BEALE | Philadelphia, Pa. |
| S. T. BEALE, JR. | Philadelphia, Pa. |
| C. S. BECK | Wilkesbarre, Pa. |
| G. V. BLACK | Jacksonville, Fla. |
| C. F. W. BODECKER | New York, N.Y. |
| E. A. BOGUE | New York, N.Y. |
| W. G. A. BONWILL | Philadelphia, Pa. |
| C. A. BRACKETT | Newport, R. I. |
| THOMAS BRADLEY | New York, N.Y. |
| EDWARD C. BRIGGS | Boston, Mass. |
| ALBERT H. BROCKWAY | Brooklyn, N.Y. |
| A. E. BROWN | Chicago, Ill. |
| WM. CARR | New York, N.Y. |
| GEO. H. CHANCE | Portland, Oregon. |
| G. F. CHENEY | St. Johnsbury, Vt. |
| DWIGHT M. CLAPP | Boston, Mass. |
| JOHN T. CODMAN | Boston, Mass. |
| CHAS. D. COOK | Brooklyn, N.Y. |
| J. N. CROUSE | Chicago, Ill. |
| EDW. T. DARBY | Philadelphia, Pa. |
| H. S. DRAPER | Boston, Mass. |
| WM. H. DWINELLE | New York, N.Y. |
| A. R. EATON | Elizabeth, N.J. |
| ALBERT T. EMERY | Boston, Mass. |
| CHAS. J. ESSIG | Philadelphia, Pa. |
| J. N. FARRER | New York, N.Y. |
| T. FILLEBROWN | Portland, Me. |
| W. B. FINNEY | Baltimore, Md. |
| T. A. FLETCHER | New York, N.Y. |
| M. W. FOSTER | Baltimore, Md. |
| CHAS. E. FRANCIS | New York, N.Y. |
| E. C. FUNDENBERG | Pittsburg, Pa. |
| W. F. FUNDENBERG | Pittsburg, Pa. |
| W. H. FUNDENBERG | Pittsburg, Pa. |
| E. S. GAYLORD | New Haven, Conn. |
| J. P. GERAN | Brooklyn, N.Y. |
| A. H. GILSON | Boston, Mass. |
| S. H. GUILFORD | Philadelphia, Pa. |
| JOHN I. HART | New York, N.Y. |
| O. E. HILL | Brooklyn, N.Y. |
| J. F. P. HODSON | New York, N.Y. |
| J. MORGAN HOWE | New York, N.Y. |
| ROBERT HUEY | Philadelphia, Pa. |
| A. O. HUNT | Iowa City, Iowa. |
| LOUIS JACK | Philadelphia, Pa. |
| V. H. JACKSON | New York, N.Y. |
| C. R. JEFFERIS | Wilmington, Del. |
| *C. A. KINGSBURY | Philadelphia, Pa. |
| W T. LA ROCHE | Harrington Park, N.J. |
| W. K. LEECH | Philadelphia, Pa. |
| *FRED. A. LEVY | Orange, N.J. |
| WILBUR F. LITCH | Philadelphia, Pa. |
| J. BOND LITTIG | New York, N.Y. |
| BENJAMIN LORD | New York, N.Y. |
| GEO. W. LOVEJOY | Montreal, Ont. |
| JOHN S. MARSHALL | Chicago, Ill. |
| H. J. MCKELLOPS | St. Louis, Mo. |
| CHARLES MCMANUS | Hartford, Conn. |
| JAMES MCMANUS | Hartford, Conn. |
| DAN'L NEAL MCQUILLAN | Philadelphia, Pa. |
| FREDERIC A. MERRILL | Boston, Mass. |
| HORATIO C. MERIAM | Salem, Mass. |
| W. D. MILLER | Berlin, Germany. |
| GEO. T. MOFFATT | Boston, Mass. |
| A. L. NORTHROP | New York, N.Y. |
| W. E. PAGE | Boston, Mass. |
| S. B. PALMER | Syracuse, N.Y. |
| C. B. PARKER | Brooklyn, N.Y. |
| D. D. PEABODY | Stoneham, Mass. |
| C. N. PEIRCE | Philadelphia, Pa. |
| S. G. PERRY | New York, N.Y. |
| W. A. PHREANER | Philadelphia, Pa. |
| CHAS. E. PIKE | Philadelphia, Pa. |
| W. E. PINKHAM | New York, N.Y. |
| W. H. POTTER | Boston, Mass. |
| CHAS. P. PRUYN | Chicago, Ill. |
| H. C. REGISTER | Philadelphia, Pa. |
| F. A. ROY | New Orleans, La. |
| Z. T. SAILER | New York, N.Y. |
| L. D. SHEPARD | Boston, Mass. |
| GEO. R. SHIDLE | Pittsburg, Pa. |
| EUGENE H. SMITH | Boston, Mass. |
| B. HOLLY SMITH | Baltimore, Md. |
| H. A. SMITH | Cincinnati, Ohio. |
| S. G. STEVENS | Boston, Mass. |
| W. X. SUDDUTH | Minneapolis, Minn. |
| EUGENE S. TALBOTT | Chicago, Ill. |
| *AMBLER TEES | Philadelphia, Pa. |
| J. D. THOMAS | Philadelphia, Pa. |
| JAMES TRUMAN | Philadelphia, Pa. |
| WM. H. TRUEMAN | Philadelphia, Pa. |
| W. W. WALKER | New York, N.Y. |
| I. F. WARDWELL | Stanford, Conn. |
| G. W. WARREN | Philadelphia, Pa. |
| T. S. WATERS | Baltimore, Md. |
| GEO. W. WELD | New York, N.Y. |
| JULIUS G. W. WERNER | Boston, Mass. |
| JACOB L. WILLIAMS | Boston, Mass. |
| *R. B. WINDER | Baltimore, Md. |
| C. A. WOODWARD | New York, N.Y. |
| J. A. WOODWARD | Philadelphia, Pa. |
| W. A. WOODWARD | New York, N.Y. |
| W. J. YOUNGER | San Francisco, Cal. |

**Deceased.*

The officers of the INTERNATIONAL DENTAL PUBLICATION COMPANY serve without salary, the investments of the stockholders merely guarantee the success of the enterprise, ALL PROFITS being devoted to the enlargement or improvement of the INTERNATIONAL DENTAL JOURNAL.

**The support of every dentist having the welfare of his profession at heart is earnestly solicited. Subscription, $2.50 per Annum.**

***THE INTERNATIONAL DENTAL PUBLICATION CO.***

